# The Risk of Retinopathy of Prematurity in the Infants following Assisted Reproductive Technology: A Meta-Analysis

**DOI:** 10.1155/2019/2095730

**Published:** 2019-07-11

**Authors:** Lixiong Gao, Weiyang Shao, Na Li, Chunyu Tian, Hongzhen Jia, Xiujun Peng, Qian Shi

**Affiliations:** Ophthalmology Department, The 6th Medical Centre of Chinese People's Liberation Army General Hospital, Beijing 100048, China

## Abstract

Currently, the use of assisted reproductive technology (ART) is increasing. Because of the poor prognosis of retinopathy of prematurity (ROP), the association between ART and the ROP has been explored in several studies, but the result was still inconclusive. Conducting a meta-analysis, we evaluated the risk of ROP in relation to the ART. Subgroup analysis as well as groups with different embryo numbers and different ROP stages was further analyzed. The PubMed, Embase, and Cochrane Library databases were searched for studies recording data about both the use of ART and ROP occurrence simultaneously. Odds ratios (ORs) and 95% confidence interval (95%CI) were calculated to analyze the association by using random- or fixed-effect models based on heterogeneity test. In total 15 observational studies containing 10392 ART cases and 39474 spontaneous conception cases were included. Results showed that there was a significant association between the use of ART and ROP occurrence in the offspring (OR = 1.34, 95% CI: 1.05 to 1.73, P = 0.02). With subgroup analysis, we found that the influence actually came from a subgroup of ART, the* in vitro* fertilization (IVF). Moreover, there was a significant association between ART and ROP in singletons. Though insignificant, the ORs were larger than 1 in the analysis between ART and stage 1 and 2 ROP. But ART showed significant association with stage 3 ROP. Our study preliminarily indicated that the use of IVF was associated with higher risk of ROP occurrence. And ART is more likely to result in severe ROP and ROP in singletons. Further specific, high-quality studies with large sample size are still needed to draw more precise conclusion.

## 1. Introduction

Retinopathy of prematurity (ROP) is a proliferative vitreoretinopathy that affects premature infants, which becomes a worldwide leading cause of childhood blindness [1]. As premature birth is the main cause of ROP, the incidence of any stage ROP in United States was 68% among infants weighing <1251 g [2]. It is estimated that over 184,700 babies of 14.9 million preterm infants developed any stage of ROP, 20,000 of whom became blind or severely visually impaired [3]. Current treatments of ROP include laser photocoagulation, anti-VEGF injection and vitreous surgery in late stage ROP infants [4–6]. These treatments still rely on the timely discovery of the disease, which brings screening of ROP to a primary intervention [7].

According to the WHO, infertility has become the consequence of a disease process and will become more and more popular in 21st century [8]. Currently, there are three main therapeutic strategies in handling infertility including pharmacological therapy, surgical therapy, and assisted reproductive technology (ART) [9]. The ART was prosperous in recent decades and was able to result in birth from infertility couples who could not be treated before. Defined as all treatments or procedures that include the* in vitro* handling of both human oocytes and sperm or of embryos for the purpose of establishing a pregnancy [10], ART mainly includes* in vitro *fertilization (IVF), intracytoplasmic sperm injection (ICSI), zygote intrafallopian transfer (ZIFT), gamete intrafallopian transfer (GIFT), artificial insemination, and so forth [11]. As this nonnatural technology has become increasingly common, the related complications need to be taken into concerns. There showed evidence that ART may lead to an increased risk of low birth weight, preterm delivery, birth defects, and genetic imprinting disorders [12, 13]. The former two complications are known high risk factors for ROP [14]. As a consequence, several observational studies have explored the relationship between the use of ART and the ROP occurrence in offspring. However, the results are inconsistent. The study of Chan et al. showed that ART was associated with severe ROP [15], while Friling et al. had found that ART did not appear to be a risk factor for ROP [16]. Simultaneously, Chiarelli et al. discovered that though statistically insignificant, ART still presented higher ROP occurrence than spontaneous conception (SC) [17]. In 2018, Trifonova et al. had conducted a systematic review about studies regarding ART as an independent risk factor for ROP [18]. Results showed no significant relationship of ART and stage 3 ROP and no further meta-analysis had been performed.

As these studies have contradicted with each other, currently no study has examined the general risk of ROP in offspring in relation to their exposure to ART versus SC. In addition to the increasing application of ART together with the poor prognosis of ROP, therefore, we performed this meta-analysis to systematically assess the association between the use of ART and ROP risk. Also, the relationship of these two aspects had been further analyzed in group with different embryo numbers as well as different ROP stages.

## 2. Methods

### 2.1. Search Strategy

**Figure 1:**
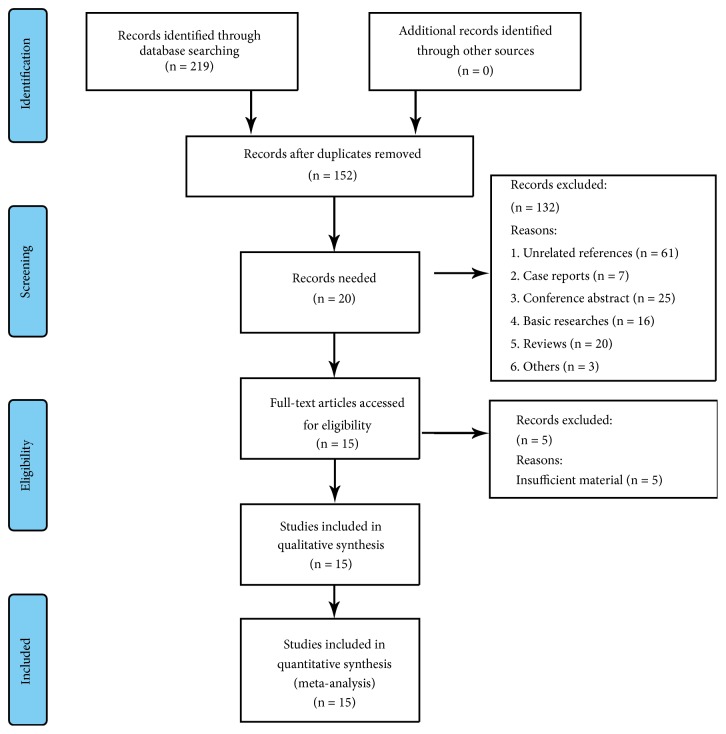
Flow chart of the literature search.

This study was conducted according to the Cochrane Handbook for Systematic Reviews and Meta-Analysis (PRISMA) guidelines. The following 3 databases were screened to complete the study: PubMed, Embase, and the Cochrane Library, up to December 2018. Keywords for ART include oocyte, fertilization, infertility, assisted reproductive technologies, assisted reproductive technology, intracytoplasmic sperm injection,* in vitro* fertilization (embryo) transfer, gamete (zygote) intrafallopian transfer, tubal embryo transfer, gamete (embryo) cryopreservation, oocyte (embryo) donation, gestational surrogacy, IVF, ICSI, and ART. Keywords for ROP include retinopathy of prematurity, ROP, retrolental fibroplasia, prematurity retinopathy, and prematurity retinopathies. The search process was conducted by two independent authors. The literature selections are shown in the PRISMA flow diagram in Figure 1.

### 2.2. Inclusion and Exclusion Criteria

Studies were considered eligible if they accord with the following criteria: (1) clinical studies focusing on both ART and ROP; (2) the association between ART and ROP should be discussed; (3) studies from which the effect estimates could be extracted or calculated from available data. The exclusion criteria were (1) unrelated references, case reports, reviews, comments, basic researches, and conference abstract; (2) studies with insufficient information.

### 2.3. Data Extraction and Assessment of Study Quality

We use a standard data extraction form to extract data; the relevant data were independently extracted by two reviewers (Gao and Shao). The following aspects were considered to be extracted: first author (s), publishing date, locations carrying out study, study design, source of the study population, study period, ART type, ROP stage, and the number of cases and controls. The Newcastle–Ottawa Scale (NOS) was used to assess the quality of both case-control studies and cohort studies. The scale of Agency for Healthcare Research and Quality (AHRQ) was used to assess the quality of cross-sectional studies. The NOS contains 3 dimensions: selection, comparability, and exposure or outcome, with 8 items and a 9-star system. AHRQ scale contains 11 items and each item represents 1 star. Two reviewers conducted the assessments independently. As one study (Shah et al.) was the secondary analysis of RCTs, we regarded it as a cross-sectional study when conducting the assessment.

### 2.4. Statistical Analysis

RevMan 5.3 was used to conduct the data statistics and meta-analysis. The odds ratios (ORs) with a 95% confidence interval [CI] [19] were used as summary statistics to evaluate the association between maternal ART and the risk of ROP in our meta-analysis. Z-test was used to assess the statistical significance of ORs. In addition to total analysis, we also carried out subgroup analyses based on study design, study location, and ART subtypes. Besides, the quantitative analysis of singletons and multiples as well as the quantitative analysis between different ROP stages were conducted. Defined as the degree of difference among included studies within the meta-analysis, the heterogeneity was investigated via I-squared (I^2^) statistic and Chi-square based Q-test. Effects models selection was carried out according to the results of heterogeneity test: P > 0.10 for the Q-test and I^2^ values less than 50% suggested no obvious heterogeneity across studies and a fixed (Mantel-Haenszel) effects model was applied; otherwise, a random (DerSimonian-Laird) effects model was applied. STATA 15.0 software (StataCorp, College Station, TX, USA) was used to conduct further statistical analysis including subgroup analysis, sensitivity analysis, funnel plots, and Egger's method. P values < 0.05 were considered statistically significant.

## 3. Results

### 3.1. The Characteristic of Studies

In total, there were 219 studies identified (Pubmed = 74; Embase = 141; Cochrane Library = 4) up to May 2019. After removing 69 duplications, 61 unrelated articles and 16 basic researches, 25 conference abstracts, 7 case reports, and 20 reviews, 23 studies were then proceeded to screening procedure. With title and abstract reading, we further eliminated 3 studies with insufficient materials and 3 references in other forms. The full texts of the rest 17 articles had been read. The study of Gocmen et al. only focused on the general manifestation of maternal and fetal outcomes of IVF and SC groups without providing the final outcome of ROP and the details of the ROP numbers in each group, which we divided into the group of insufficient materials [20]. Besides, the study of McKibbin et al. focused on the relationship between assisted conception and ROP in 1996, the relevant data was not presented in a clear table, and the description of data in the main body was ambiguous [21]. In addition to the early document with incomplete recordation of methodology which made it hard to assess the quality of the study, we also divide this article into the group of insufficient materials. In this way, remaining 15 studies were included into quantitative synthesis [15–17, 22–33]. The flow diagram was shown in Figure 1.

All 15 included articles were published between 2000 and 2019. There were 6 cohort studies, 3 case-control studies, 5 cross-sectional studies, and a secondary analysis of RCTs containing 10392 ART cases and 39474 SC cases. The characteristics of included studies were presented in Table 1. As ROP happened in premature infants, the population of studies was mainly infants with low birth weight (< 1501 g) and/or premature infants (< 32 weeks). There are 7 studies specifically focused on the IVF technology, while other studies did not distinguish many different subtypes of ART. Different ROP stages were discussed, in which 7 articles mainly focused on severe ROP (stage ≥ 3), 1 article focused on moderate to severe ROP (stage ≥ 2), 4 articles discussed all ROP stages, and 3 articles did not classify ROP stage. Those characteristics and the score of methodological quality were shown in Table 1.

### 3.2. Meta-Analysis

**Figure 2:**
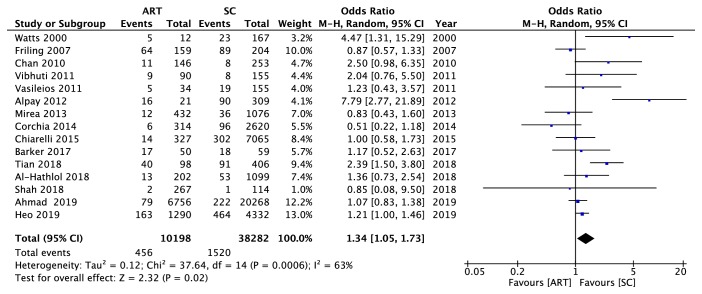
A forest plot diagram presenting the association between the use of ART and the ROP occurrence. All 3 ROP stages were generally considered. Events referred to the number of infants who developed to the ROP. Total referred to the general number of participants in the group. ART: assisted reproductive technology; SC: spontaneous conception; CI: confidence interval.

The association of ART and ROP was evaluated using the included studies. The overall results of the quantitative analysis suggested that there was a significant association between maternal ART use and the ROP occurrence in offspring (OR = 1.34, 95% CI: 1.05 to 1.73, P = 0.02) (Figure 2). A moderate heterogeneity was found (chi^2^ = 37.64, P = 0.0006, I^2^ = 63%).

**Figure 3:**
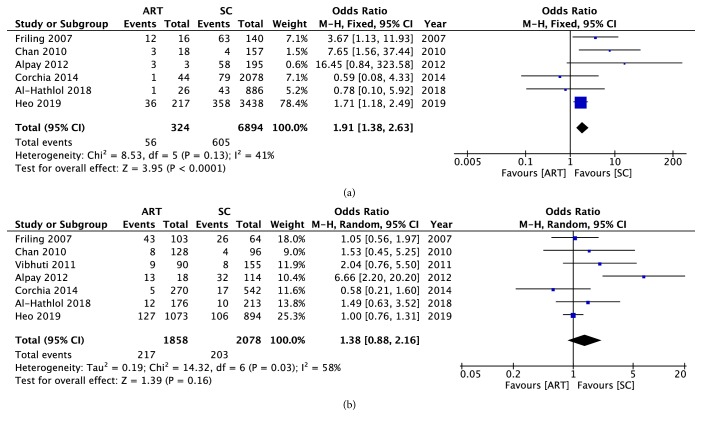
A forest plot diagram presenting the association between the use of ART and the ROP occurrence in different embryos. All 3 ROP stages were generally considered. (a) Singletons. (b) Multiples. Events referred to the number of infants who developed to the ROP. Total referred to the general number of participants in the group. ART: assisted reproductive technology; SC: spontaneous conception; CI: confidence interval.

In addition, we also evaluated the association between ART and ROP in both singletons and multiples. In singletons, there was a significant association between maternal ART use and the ROP occurrence in offspring (OR = 1.91, 95% CI: 1.38 to 2.63, P < 0.0001) (Figure 3(a)). A relatively low heterogeneity was found (chi^2^ = 8.53, P = 0.13, I^2^ = 41%). However, in multiples, there was a no significant association between ART and the ROP (OR = 1.38, 95% CI: 0.88 to 2.16, P = 0.16) (Figure 3(b)). A moderate heterogeneity was found (chi^2^ = 14.32, P = 0.03, I^2^ = 58%).

**Figure 4:**
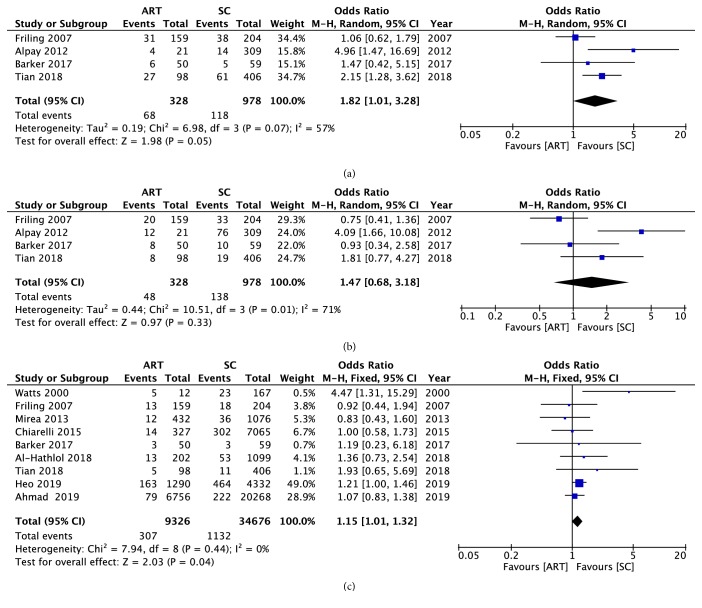
A forest plot diagram presenting the association between the use of ART and the ROP occurrence in different ROP stages. (a) Stage 1 ROP. (b) Stage 2 ROP. (c) ≥Stage 3 ROP. Events referred to the number of infants who developed to the ROP. Total referred to the general number of participants in the group. ART: assisted reproductive technology; SC: spontaneous conception; CI: confidence interval.

Besides, the relationship of ART and different ROP stages was discovered in our meta-analysis. The significance of the association between ART and the stage 1 ROP was just at the statistical border (OR = 1.82, 95% CI: 1.01 to 3.28, P = 0.05) (Figure 4(a)). A moderate heterogeneity was found (chi^2^ = 6.98, P = 0.07, I^2^ = 57%). No significant association between ART and stage 2 ROP had been found (OR = 1.47, 95% CI: 0.68 to 3.18, P = 0.33) (Figure 4(b)). A high heterogeneity was found (chi^2^ = 10.51, P = 0.01, I^2^ = 71%). A significant result had been found between ART and stage 3 ROP (OR = 1.15, 95% CI: 1.01 to 1.32, P = 0.04) (Figure 4(c)). A low heterogeneity was found (chi^2^ = 7.94, P = 0.44, I^2^ = 0%).

### 3.3. Subgroup Analysis and Corresponding Sensitivity Analysis

**Figure 5:**
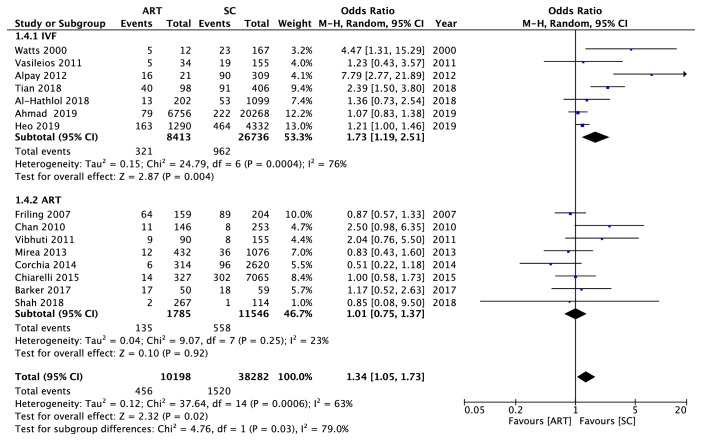
A subgroup analysis of forest plot diagram presenting the association between the use of IVF and the ROP occurrence (1.4.1 IVF) as well as the use of ART mixture (1.4.2 ART) and the ROP occurrence. All 3 ROP stages were generally considered. Events referred to the number of infants who developed to the ROP. Total referred to the general number of participants in the group. ART: assisted reproductive technology; SC: spontaneous conception; CI: confidence interval.

The subgroup analyses were conducted according to the study design and area. There were no special positive findings on the association between ART and ROP. Results were listed in Table 2. Since a number of study focused on IVF, we also performed the subgroup analysis on IVF and the rest ART types. We noticed that there was a significant association between IVF along and the risk of ROP (OR = 1.73, 95% CI: 1.19 to 2.51, P = 0.004) (Figure 5). A high heterogeneity was found (chi^2^ = 24.79, P = 0.0004, I^2^ = 76%). And in the rest ART mixture subgroup, no statistically difference had been found (OR = 1.01, 95% CI: 0.75 to 1.37, P = 0.92) (Figure 5). But the heterogeneity was low (chi^2^ = 9.07, P = 0.25, I^2^ = 23%). The results illustrated that though there appeared to be significant relation between ART use and the ROP occurrence according to the main meta-analysis, the effect actually came from the subtype of ART, the IVF. Each study in the analysis of ART subgroup and ROP was removed sequentially to verify the stable effect on our results. The result of the sensitivity analysis showed no obvious changes after excluding any study, which implied that our results were stable and reliable (Supplementary Figure 1).

### 3.4. Publication Bias

**Figure 6:**
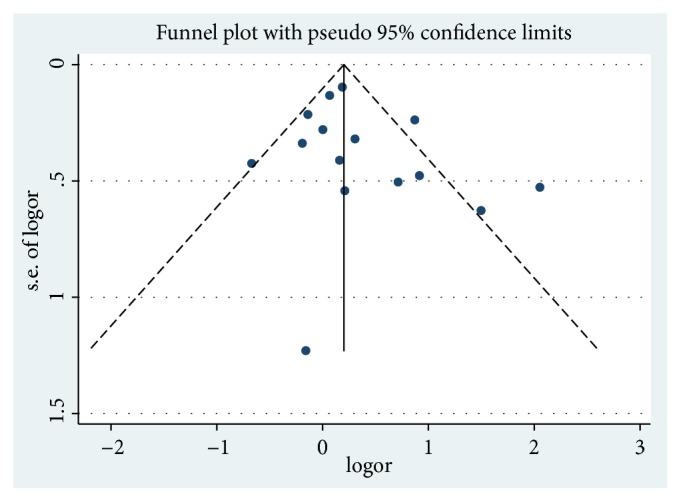
Funnel plot of studies examining the association between the use of ART and ROP occurrence. Central solid line indicated the overall estimated effect.

We performed the Egger's method to explore the underlying publication bias in our meta-analysis. The related P value was 0.271. Corresponding Egger's results and the Egger's funnel plots could be found in supplementary materials (Supplementary Figure 2). The funnel plot of all included studies was shown in Figure 6. It showed that majority of studies gathered at the top of the funnel plot (Figure 6). Simultaneously all the studies showed a relatively symmetrical distribution around the central solid line (overall estimated effect), reflecting the publication bias within the current meta-analysis was low (Figure 6).

## 4. Discussion

As both the increasing adoption of ART and the poor prognosis of ROP become major concerns, it is necessary to discover the relationship between these two aspects. This meta-analysis quantitatively evaluated the association between the use of ART and the general risk of ROP as well as the association between ART and ROP in groups with different embryo numbers and different ROP stages. In this study, 49866 patients within 15 observational studies were included to explore the relationship between ROP occurrence and the use of ART. Significant effect had been found in ART group, compared with SC group. Moreover, with subgroup analysis, we further found that the difference actually existed within the subgroup of ART, the IVF. Besides, we also found significant association between ART and ROP in singletons, which was insignificant in multiples. The relationship between ART and different ROP stages was also discovered. The values of OR in all three ROP stage were larger than 1. There was a significant association between ART and stage 3 ROP. Current findings have improved the understanding of the relation between ART and ROP, provided evidence of the underlying complications of IVF. In this way, doctors should inform couples the underlying risks of ROP occurrence (especially stage 3 ROP) who decide to adopt the IVF technology. Also, pediatric ophthalmologist should pay more attention to those IVF babies when performing ROP screening. More important, to avoid serious visual complications, doctors may advise couples who have gave babies with IVF to conduct ROP screening routinely.

In 2018, Trifonova et al. have published a systematic review to discuss the relationship between ART and ROP. Consistent with our findings, the results showed that though insignificant, ROP was observed which more frequently happened in ART [18]. As this systematic review only included studies directly comparing ROP happened in ART and SC groups, the observational studies which contained the data of ROP when comparing the birth defects between ART and SC could be omitted. In our meta-analysis, we included all studies containing available data which could be used in further quantitative analysis. Moreover, subgroups of ART, different number of embryos, and different ROP stages could also be discussed.

According to the incomplete statistics, there were over 1643456 ART cycles being initiated all over the world in 2010, accounting for 474 cycles per million populations. From 2008 to 2010, the annual increase rate of ART is over 10% [34]. An ART report from the European Society of Human Reproduction and Embryology showed that the percentage of IVF in total ART was 20.0% and the percentage of ICSI was 46.3% [35]. In the current study, IVF was confirmed to be significantly associated with ROP. As ROP can be viewed as an arrest of normal retinal vascular development in the preterm infant, with subsequent pathological compensation that result in aberrant vascularization, premature birth is the core risk factor in ROP [36]. A number of studies had proved that use of IVF may lead to premature birth [37]. One of the reasons could be the use of autologous or thawed oocyte. As autologous women are super-ovulated, hormonal regimens could lead to disturbed endometrial maturation, resulting in abnormal implantation [38]. Besides, the maternal age, type of infertility, chromosomal anomalies oriented from* in vitro* manipulation, epigenetic disorders including abnormal methylation, cryopreservation of gamete or embryo, and* in vitro* culture environment, all these aspects may specifically produce influence on the ending effect of ART, compared with SC [39], which could potentially raise the possibility of preterm birth. In addition, due to the advances in healthcare and technology, the number of survived preterm infants increased these years. Besides, Hansen et al. had found the increasing risk of birth defect following IVF/ICSI [40]. As the risk for birth defects in ICSI is similar to that in IVF [41] and ICSI was the progress of IVF, we may deduce that ICSI could also possibly build significant connections to the ROP. Taken together, the underlying mechanism that IVF/ICSI may be a risk factor of ROP is due to the premature birth caused by IVF/ICSI, which should raise an alarm to couples planning to perform IVF/ICSI.

In the current study, interestingly we found that, compared with multiple group, ART showed significant relationship with ROP in singleton group, which contradicted with the notion that multiple-birth infants had higher risk of prematurity [42]. Friling et al. attributed this difference to the lower gestational age and body weight of singletons following IVF [16]. Study have proven that compared with SC, ART singleton pregnancies had a significant increase risk of preterm birth, very preterm birth, low birth weight, and very low birth weight [43, 44]. Besides, it was showed that, among ART children, there were more low birth weights or very low birth weight children in singleton birth, compared with multiples [45]. However, Chambers et al. found that, among ART children, there were more multiples than singletons being conceived, and it showed much higher probability for multiples to become preterm birth or even stillborn (over dozens to several hundred times) [46]. As the preterm birth in children born with ART showed inconsistent results among these studies, the biochemical mechanism of the significant associations between ART and ROP in singletons still requires further experiments to discover. Since singleton birth is gradually increasing these years [35], it is worth noting that when performing IVF to gestate singletons, couples and doctors need to take the risk of ROP occurrence into deliberate consideration.

The relationship between ART and different ROP stages were also discovered in this study. Though insignificant results were presented in both stage 1 and 2 ROPs, the ORs were larger than 1, which corresponded to the results of the main meta-analysis. However, there was a significant association between ART and stage 3 ROP, giving the notion that ART could lead to the severe ROP and bring in irreversible visual impairment. Amount of studies has proved that ART accounts for a high proportion of severe ROP [15, 47]. Besides, the P value was just at the 0.05 level in stage-1 analysis. When more studies are included in the future, it could be believed that ART would show significant relationship to stage-1 ROP. As hitherto there were more studies focusing on stage 3 ROP than early ROP stages, this inconsistency might due to the limited researches regarding different ROP stages. To clearly illustrate the underlying mechanism and to fully discover the certain relationship between ART and different ROP stages, more precise and well-designed studies should be performed.

In this study, IVF was significantly linked with ROP. However, current results showed that there was no significant relationship between the rest ART group and ROP. There were several possible reasons. First, IVF mainly contain two methods: the classic IVF without micromanipulation and the ICSI [31]. These two methods occupied over 60% of ART [35], leaving a relative small portion for the rest ART methods, which could bring bias to the results. Second, compared with other ART methods, conducting IVF requires many in vitro procedures, which is more likely for gametes or fused zygotes to get harm. Third, mothers who conceive via IVF are likely to have more thorough prenatal screenings and lifestyle modifications, such as smoking cessation and weight loss, which are possibly associated with infertility [32]. The maternal state difference between IVF and the rest ART methods could also be an important reason. However, more specific clinical studies should be carried out to draw precise conclusion.

There was obvious heterogeneity within the current meta-analysis. The subgroup analysis was not able to specifically locate the origin of the heterogeneity. As the data in the included articles were only a part of the study or indirect comparison, the heterogeneity could be derived from the confounding. However, when IVF was removed, the heterogeneity decreased significantly in the meta-analysis, indicating that the heterogeneity could be partly come from IVF group. On the other hand, IVF group showed a high heterogeneity. Nevertheless, the 7 IVF studies all presented ORs larger than 1, illustrating that IVF was significantly associated with ROP though heterogeneity existed. Besides, with sensitivity analysis, we found the significant association between IVF and ROP was stable and reliable, which increased our confidence to draw the conclusion. Moreover, potential publication bias played an important role on the analysis. With Egger's method, we observed little evidence of publication bias in the current study.

There were several limitations in this study. First, due to the limited number of studies as well as the indirect data included, the meta-analysis was performed insufficiently, which decreased the quality of evidence. Second, the uncontrolled residual confounding was not able to be excluded. Maternal factors, complexity of ART treatment, and different use or screening standard render the specification of individual factors extremely challenging, which brings potential bias to the study. Third, the biological mechanism underlying the relationship between using ART and the risk of ROP in offspring cannot be specified in the current study. Therefore, more studies directly comparing the relationship between ART and ROP should be included in future reviews. Besides, the subtype of ART within studies should also be further distinguished.

## 5. Conclusion

In summary, there was significant relationship between ART and ROP in the offspring. Specifically, the relationship mainly came from the subtype of ART, the IVF. Moreover, ART was significantly associated with ROP in singletons and stage 3 ROP. Due to the limited number of studies, there should be more studies with direct comparing data and detailed subtype information to draw more precise conclusion in future reviews. Corresponding molecular mechanism may be explored in future studies.

## Figures and Tables

**Table 1:** Characteristics of the included studies.

Author (year)	Country	Study design	Source of the study population	Study period	ART type	ROP stage	Case/ control	Methodological quality
Watts et al. (2000)	United Kingdom	Cross-sectional study	Infants fulfilled the Royal College of Ophthalmologists and British Association of Perinatal Medicine criteria for screening for ROP	1995-1998	IVF	ROP (stage ≥3)	21/158	4

Friling et al. (2007)	Israel	Case-control study	Premature infants with birth weight < 1500 g	1998-2000	ART	ROP (stage 0, 1, 2, 3)	159/204	6

Chan et al. (2010)	United States	Cross-sectional study	Infants with GA < 32 weeks and/or birth weight < 1500 g	2002-2007	ART	Lasered or post GA lasered	146/253	6

Vibhuti et al. (2011)	Canada	Cohort	Preterm multiple births born at ≤ 32 weeks GA	2005-2008	ART	ROP (stage >2)	137/233	5

Vasileios et al. (2011)	Greece	Cross-sectional study	Preterm neonates born with a GA of 24–32 weeks or birth weight < 1500 g and hospitalised in the NICU	2000-2008	IVF	ROP	34/189	8

Alpay et al. (2012)	Turkey	Cross-sectional study	Premature infants with GA ≤ 34 and examined for ROP	2005-2009	IVF	ROP (stage 1, ≥2)	21/309	7

Mirea et al. (2013)	Canada	Case-control study	Twin pairs born at 32 weeks gestation with both co-twins admitted to a Level 3 NICU	2010-2011	ART	ROP (stage ≥3)	432/1076	7

Corchia et al. (2014)	Italy	Cohort	Infants born at 22 to 31 completed weeks of GA and admitted to NICU	2003-2005	ART	ROP (stage ≥3)	314/2620	8

Chiarelli et al. (2015)	Canada	Cross-sectional study	Singleton infants born at 23 to 32 weeks GA	2010-2012	ART	ROP (stage ≥3)	327/7065	6

Barker et al. (2017)	United Kingdom	Case-control study	Infants born < 32 weeks and/or < 1501 g in Homerton University Hospital NHS Founda on Trust	2007-2011	ART	ROP (stage 1, 2, 3)	50/59	6

Shah et al. (2018)	United States	Secondary analysis of RCTs	Multiple gestation pregnancies in 14 academic sites across the United States	2004-2006	ART	ROP	89/38	6

Tian et al. (2018)	China	Cohort	Very low birth weight infants admitted at West China Second University Hospital	2012-2015	IVF	ROP (stage 1, 2, ≥3)	98/406	3

Al-Hathlol (2018)	Kingdom of Saudi Arabia	Cohort	Low birth weight preterm infants admitted in NICU of King Abdulaziz Medical City	2000-2014	IVF	ROP (stage 3-4)	236/1297	6

Heo et al. (2019)	Korea	Cohort	Very low birth weight infants infant admitted or transferred within 28 days after birth to NICU	2014-2016	IVF	ROP (stage ≥3)	1572/5299	7

Ahmad et al. (2019)	United States	Cohort	Infants from Pediatrix-managed NICUs delivered between 23 and 34 weeks GA	2009-2016	IVF	ROP (stage ≥3)	6756/20268	6

**Table 2:** Summary of subgroup analysis of the meta-analysis results. RE: random effects; FE: fixed effects. IVF: in vitro fertilization; ART: assisted reproductive technology.

Groups	Studies	Test of association				Heterogeneity		
		OR [95%CI]	p value	Model	Z	*X* ^2^	p value	*I* ^2^(%)
Total studies	15	1.34 [1.05-1.73]	0.02	RE	2.32	37.64	0.001	63

*Subgroup analysis*								

*Study design*								

Cohort	7	1.28 [0..96-1.70]	0.09	RE	1.69	14.45	0.025	59

Case-control	3	0.9 [0.65-1.25]	0.53	FE	0.62	0.51	0.777	0

Cross-sectional	5	2.39 [1.07-5.33]	0.03	RE	2.13	15.17	0.004	74

*Region*								

Asia	4	1.33 [0.92-1.91]	0.13	RE	1.51	10.47	0.015	71

Europe	5	1.83 [0.7-4.82]	0.22	RE	1.23	19.75	0.001	80

America	6	1.10 [0.90-1.36]	0.34	FE	0.94	5.37	0.372	7

*IVF and ART*								

IVF	7	1.73 [1.19-2.51]	0.004	RE	2.87	24.79	0.004	76

ART	8	0.97 [0.76-1.23]	0.8	FE	0.25	9.07	0.247	23

## Data Availability

All the data used to support the findings of this study are available from the corresponding author upon request

## Supplementary Materials

Supplementary Materials Supplementary Figure 1: the sensitivity analysis of the main meta-analysis. Supplementary Figure 2: the summary sheet and the funnel plot of Egger's method.
