# Supplementary material for: The Risk of Retinopathy of Prematurity in the Infants following Assisted Reproductive Technology: A Meta-Analysis
Source: Biomed Res Int. 2019 Jul 11;2019:2095730. doi: 10.1155/2019/2095730 (PMC6657639; doi:10.1155/2019/2095730)
Supplement: Supplementary Materials — Supplementary Figure 1: the sensitivity analysis of the main meta-analysis. Supplementary Figure 2: the summary sheet and the funnel plot of Egger's method. [file 2095730.f1.pdf]

Supplementary Materials

The risk of retinopathy of prematurity in the infants following assisted reproductive technology: a meta-analysis

Lixiong Gao[1], Weiyang Shao[1], Na Li[1], Hongzhen Jia[1], Chunyu Tian[1], Xiujun Peng[1], Qian Shi[1*]

[1] Ophthalmology Department, the 6th Medical Centre of Chinese People's Liberation Army General Hospital, Beijing, 100048, China

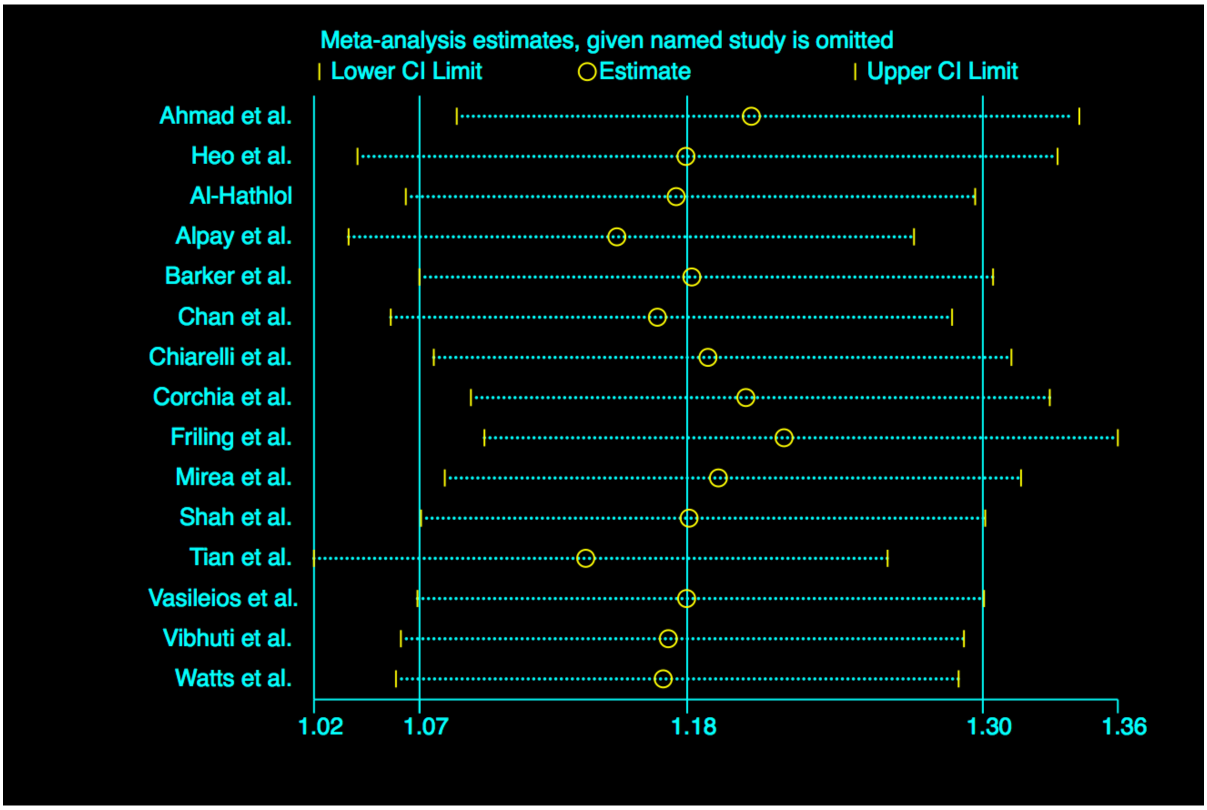

Supplementary Figure 1 The sensitivity analysis of the main meta-analysis. Each estimate with its 95% CI was the results after omiting the corresponding study. It showed that all estimates were located within the general 95% CI (1.07-1.30). CI: confidence interval.

a

```
Egger's test for small-study effects:
Regress standard normal deviate of intervention
effect estimate against its standard error

Number of studies =   15                          Root MSE      =    1.621
```

Std_Eff	Coef.	Std. Err.	t	P>|t|	[95% Conf.	Interval]
slope	.0392259	.1727857	0.23	0.824	-.3340548	.4125067
bias	.8337919	.7252938	1.15	0.271	-.73311	2.400694

```
Test of H0: no small-study effects          P = 0.271
```

b

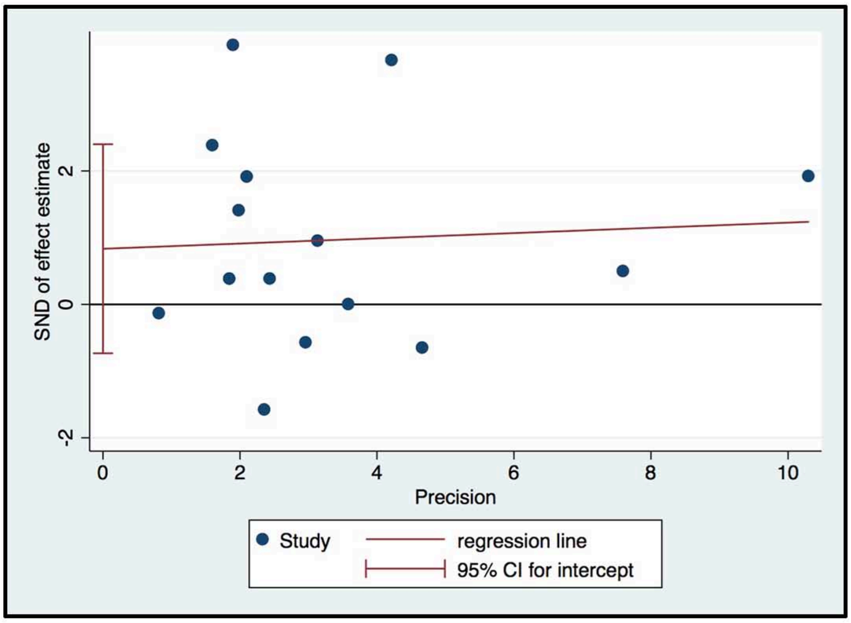

Supplementary Figure 2 The summary sheet and the funnel plot of Egger's method. a: summary sheet; b: funnel plots.
